# The complete chloroplast genome sequence of *Phyllagathi hainanensis* (Melastomataceae) and phylogenetic analysis

**DOI:** 10.1080/23802359.2021.2002202

**Published:** 2021-11-23

**Authors:** Ai-Fang Weng, Lang-Xing Yuan, Qing-Hui Sun, Hong-Xin Wang

**Affiliations:** a Zhai Mingguo Academician Work Station, Sanya University, Sanya, China; b Institute Arts, Sanya University, Sanya, China; c School of Tropical Medicine and Laboratory Medicine, Hainan Medical University, Haikou, China

**Keywords:** *Phyllagathi hainanensis*, chloroplast genome, phylogenetic tree, Melastomataceae

## Abstract

*Phyllagathi hainanensis* (Merr. et Chun) C. Chen is a small shrubs of Melastomataceae. It is only distributed in Hainan provinces of China. The complete chloroplast genome of *P. hainanensis* is reported in this study. The complete chloroplast genome of *P. hainanensis* is 156,123 bp in length with a typical quadripartite structure, consisting of a large single-copy region (LSC, 85,497 bp), a single-copy region (SSC, 17,076 bp), and a pair of inverted repeats (IRs, 26,775 bp). There are 129 genes annotated, including 37 transfer RNA genes, 8 ribosomal RNA genes, and 84 proteincoding genes. The complete plastome sequence of *P. hainanensis* will provide a useful resource for phylogenetic studies in Melastomataceae.

*Phyllagathis hainanensis* (Merr. et Chun) C. Chen belongs to Melastomataceae. The *Phyllagathis* is distributed mainly in the tropical and subtropical regions of Asia (Cellinese 2002; Chen and Renner 2007; Lin et al. 2017).There are 25–28 species of *Phyllagathis* in China (Chen 1984; Chen and Renner 2007). *P. hainanensis* is an endemic species to Hainan and occurs in sparse to the forests, mountain slopes, and hillsides with an altitude of 600–1400 m (Lin et al. 2015). The most recent analyses revealed that *Phyllagathis* is not monophyletic, showing close relationships with *Allomorphia* Blume, *Blastus* Lour., *Fordiophyton* Stapf, *Oxyspora* DC., *Plagiopetalum* Rehder, *Sonerila* Roxb., and *Tigridiopalma* C. Chen (Zeng et al. 2016; Zhou et al. 2019). At present, ca. 40 complete chloroplast genomes of *Phyllagathis* were all reported, the complete chloroplast genome of *P. hainanensis* is unknown. In this study, we annotated the chloroplast genome of *P. hainanensis* into GenBank public database with the accession MZ450795.

In this study, the fresh leaves of *P. hainanensis* were collected from Murui Mountain in Hainan province (110. 29°N, 19. 24°E, elevation 680 m). Voucher specimens were deposited in the herbarium of Sanya University (collector and collection number: Lang-xing Yuan, HNJXC1) .Genomic DNA of *P. hainanensis* leaves was extracted according to CTAB method (Doyle and Doyle 1987). Paired-end (PE) reads of 150 bp was conducted on an Illumina Hiseq-2500 platform at BGI-Shenzhen. Approximately, 4 GB raw data (32,192,787 Clean Reads) was generated and deposited in Sequence Read Archive (SRA) under accession number SRR14891646. China. The complete chloroplast genome was assembled by GetOrganelle. We used the pipeline PGA to performe gene annotation with *P. sessilifolia* (MK994926) as a reference. Analysis of boundaries between IRs and single copy regions was performed by online program GeSeq (Tillich et al. 2017).

The plastome of *P. hainanensis* was found to possess total length 156,123 bp with the typical quadripartite structure containing two inverted repeats (IRs) of 26,775 bp, a large single-copy (LSC) region of 85,497 bp and a small single-copy (SSC) region of 17,076 bp. There were 129 genes, including 37 transfer RNA genes, 8 ribosomal RNA genes, and 84 proteincoding genes. The total GC content of chloroplast genome in *P. hainanensis* was 36.98%.

We used RAxML (Stamatakis 2006) with 1000 bootstraps under the GTRGAMMAI substitution model to reconstruct a maximum-likelihood (ML) phylogeny of 13 published complete plastomes of *Phyllagathis*, using *Astronia smilacifolia* (MK994883.1) and *Memecylon ligustrifolium* (MK994913.1) as outgroups (Figure 1). Our analyses show that *P. hainanensis* is sister relationship with the remaining species of this genus. Our results provide valuable data and shed light on the phylogenomic study of Melastomataceae.

**Figure 1.**
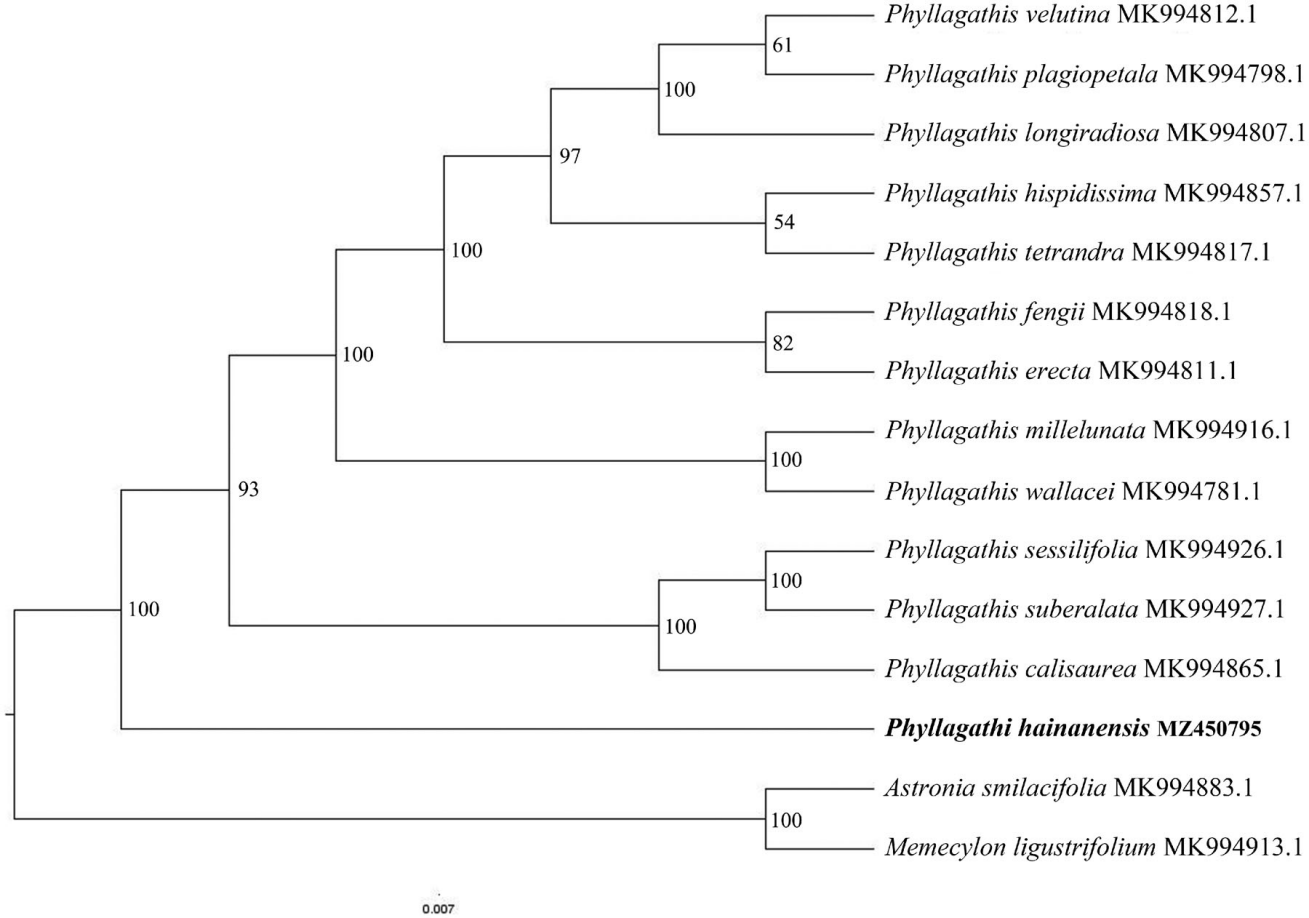
Phylogenetic tree based on 15 complete sequences of chloroplast genome in different species. The accession number in bold font was the newly sequenced *Phyllagathi hainanensis* in this study.

## Data Availability

The genome sequence data that support the findings of this study are openly available in GenBank of NCBI at https://www.ncbi.nlm.nih.gov/ under the accession MZ450795. Raw Illumina data were deposited in the NCBI Sequence Read Archive (SRA: SRR14891646, BioProject: PRJNA739828, and Bio-Sample: SAMN19837121).
